# RPN2 is effective biomarker to predict the outcome of combined chemotherapy docetaxel and cisplatin for advanced gastric cancer

**DOI:** 10.18632/oncotarget.24622

**Published:** 2018-03-08

**Authors:** Daisuke Fujimoto, Takanori Goi, Kenji Koneri, Yasuo Hirono

**Affiliations:** ^1^ Department of Surgery 1, Faculty of Medicine, University of Fukui, Fukui, Japan

**Keywords:** RPN2, gastric cancer, docetaxel, cisplatin, predictive biomarker

## Abstract

Preoperative chemotherapy, often using docetaxel and cisplatin, is a famous treatment option for advanced gastric cancer in Japan. But, there are no effective biomarkers that predict the therapeutic outcome on gastric cancer. Ribophorin II (RPN2) silencing, which decreases glycosylation of P-glycoprotein (P-gp) and membrane localization, restores the sensitivity to docetaxel and cisplatin. We inquired whether RPN2 expression in advanced gastric cancer biopsy tissues may be a predictive biomarker for docetaxel and cisplatin combination preoperative chemotherapy.

We judged RPN2 expression immunohistochemically in upper endoscopic biopsy tissues from 40 advanced gastric cancer patients, who received the combination preoperative chemotherapy of docetaxel and cisplatin and gastrectomy with D2 resection during 2008-2014, and compared clinicopathological effects between RPN2-positive and RPN2-negative groups. We also examined sensitivity of RPN2-knockout gastric cancer cells by genome editing to docetaxel and cisplatin.

RPN2 expression was observed in 19 of 40 gastric cancer cases. The RPN2-negative group had better clinicopathological responses to docetaxel and cisplatin combination chemotherapy than the RPN2-positive group, especially, in assessment of the histopathological criteria to preoperative chemotherapy. And RPN2-negative group had a significantly higher overall survival and progression-free survival compared to the RPN2-positive group. We also found RPN2-knockout to change docetaxel and cisplatin sensitivity *in vitro*.

RPN2 expression in upper endoscopic biopsy tissues can be an effective predictive biomarker for the treatment outcome to docetaxel and cisplatin combination preoperative chemotherapy in advanced gastric cancer.

## INTRODUCTION

Gastric cancer is one of the most common cancers, especially in eastern Asia [1]. Recently, even as the progress of early diagnosis and surgical techniques have improved the result of treatment for gastric cancer, the mortality rate stays high, mainly due to complex biological features of gastric cancer and its high grade malignancy [2]. Many patients with gastric cancer are diagnosed at an advanced stage and consequently have distant metastases and poor prognosis. The commonly treatment for advanced gastric cancer is to reach R0 resection. Though gastrectomy with D2 lymph node dissection, the so-called extended dissection in western countries, has been undergone, recurrence including peritoneal metastases has been found often in advanced gastric cancer. In an effort to decrease incidence of relapse after R0 resection, postoperative adjuvant chemotherapy, such as S-1, is recommended in Japan [3].

The possible benefits of preoperative chemotherapy include increasing the likelihood of curative resection by down-staging of the tumor, eliminating micrometastasis and better compliance against chemotherapy by avoiding surgery-related gastrointestinal symptoms. MAGIC trial demonstrated that preoperative chemotherapy achieved down-staging and revealed longer survival than surgery alone [4]. We think that conditions required for preoperative chemotherapy regimen include (1) high response rate in a short period of time, and (2) no affected the surgical timing by side effects. Various phase I and phase II studies have been conducted to judge combination chemotherapy of docetaxel, cisplatin and S-1 (DCS) in severe advanced gastric cancer patients, and these trials demonstrated good response rate [5–7]. Reports on Phase II trial of DCS combined chemotherapy for unresectable/recurrent gastric cancer showed that Koizumi et al., published a response rate 87.1%, median survival time 660 days; Sato et al., published a response rate 81.3%, median survival time 687 days [6, 7]. Both reported higher response rate than S-1/Cisplatin combined chemotherapy. Therefore, we selected DCS combined chemotherapy for preoperative chemotherapy regimen. But docetaxel and cisplatin combination chemotherapy is severe toxic. Consequently, if this chemotherapy is not effective to malignant tumors, its adhibition is merely senseless, but also really severe detrimental for patients. What is worse, as preoperative chemotherapy intervene curative surgical treatment, there is at risk for disease progression and of missing out the chance to heal no-responded patients of cancer. Hence, specific molecular biomarker that calculates effective response to especial drugs has possibilities of exceedingly helpful to select patients who may derive a benefit from these drugs.

Recently, downregulation of Ribophorin II (RPN2), which is part of an N-oligosaccharyl transferase complex, efficiently induced apoptosis in docetaxel-resistant human breast cancer cells in the presence of docetaxel, and RPN2 silencing repressed tumorigenicity and sensitized the tumors to cisplatin treatment [8, 9]. And high expressed RPN2 had a significantly lower 5-year survival rate and higher recurrence rate compared to the gastric cancer cases with low RPN2 expression [10]. These results indicate that RPN2 expression prospects a predictive biomarker for tolerance to combination chemotherapy docetaxel and cisplatin. There is very little new knowledge about between expression of RPN2 and tolerance to docetaxel and cisplatin in gastric cancer.

In this report, we retrospectively investigated the operability and effectivity of predicting response biomarker RPN2 expression using pre-treatment tissues of advanced gastric cancer patients to DCS therapy, with a particular focus on the potential of RPN2 expression as a predictor to choice patients who may benefit from docetaxel and cisplatin combination preoperative chemotherapy.

## RESULTS

### Patient characteristics and RPN2 expression

**Figure 1:**
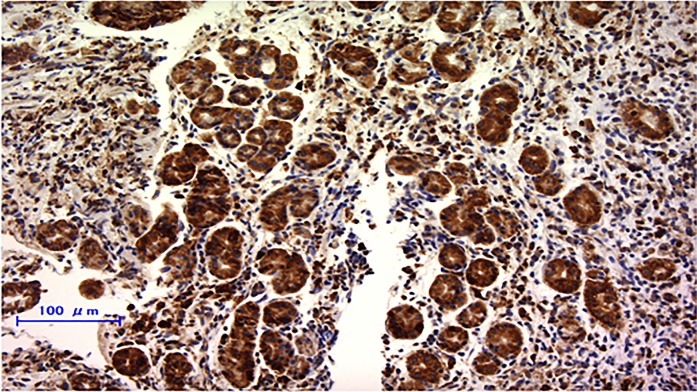
Immunohistochemically staining of RPN2 in endoscopic biopsy sample of gastric cancer RPN2 protein expression was detected in cytoplasm.

Of the 40 patients with advanced gastric cancer, we found 47.5% (19 out of 40) of patients were member of the group of RPN2 positive and 52.5% (21 out of 40) were member of the group of RPN2 negative. RPN2 expression was localized in the cytoplasm (Figure 1). No relationships could be detected between RPN2 expression and sex, age, macroscopic type, depth of tumor invasion, clinical lymph node metastasis, present of distant metastasis, and clinical stage (Table 1). But we found significant correlation between RPN2 expression and histological type only (Table 1).

**Table 1:** RPN2 expression and clinicopathological factors

Factors		RPN2 expression		P-value
		positive 19	negative 21	
Age	70≧	4	7	P = 0.488
	70<	15	14	
Sex	male	14	17	P = 0.583
	female	5	4	
cT	T3(SS)	3	4	P = 0.557
	T4a(SE)	15	17	
	T4b(SI)	1	0	
cN	N0	2	1	P = 0.897
	N1	6	6	
	N2	7	9	
	N3	4	5	
cM	absent	14	12	P = 0.333
	present	5	9	
cStage	IIB	3	0	P = 0.392
	IIIA	5	5	
	IIIB	5	4	
	IIIC	1	3	
	IV	5	9	
Macroscopic	Type 2	5	9	P = 0549
type	Type 3	7	6	
	Type 4	7	6	
Histological	differentiated	2	9	P = 0.034
type	undifferentiated	17	12	
Treatment after DCS	gastrectomy	15	16	P = 0.749
	Continue DCS, and subsequent gastrectomy	4	5	

Abbreviations: DCS = docetaxel combined with S-1 and cisplatin; RPN2 = ribophorin II. Analyzed by Fisher's exact test.

### Correlation between RPN2 expression and effects to chemotherapy

Two criteria used to judge clinical response to DCS chemotherapy showed significant differences between the RPN2-negative and the RPN2-positive groups (Table 2). Based on the RECIST v1.1 criteria, the RPN2-positive group was the result of CR: 0, PR: 6, SD: 12, PD: 1 case, and the RPN2-negative group CR: 5, PR: 13, SD: 3, PD: 0 case (P=0.002). Moreover based on the histopathological criteria, the RPN2-positive group was the result of grade3: 0, grade2: 3, grade1b: 6, grade1a: 8, grade0: 2 case, and the RPN2-negative group grade3: 5, grade2: 10, grade1b: 5, grade1a: 1, grade0: 0 case (P=0.006).

**Table 2:** Correlation between RPN2 expression and response to chemotherapy

Therapeutic efficacy		RPN2 expression		P-value
		positive 19	negative 21	
Clinical response^*^	CR	0	5	P = 0.003
	PR	6	13	
	SD	12	3	
	PD	1	0	
Histopathology	grade0	2	0	P = 0.006
	grade1a	8	1	
	grade1b	6	5	
	grade2	3	10	
	grade3	0	5	

Abbreviations: CR = complete response; PR = partial response; SD = stable disease; PD = progressive disease ^*^RECIST v1.1 Analyzed by Fisher's exact test.

### Overall survival and progression-free survival in advanced gastric cancer patients

**Figure 2:**
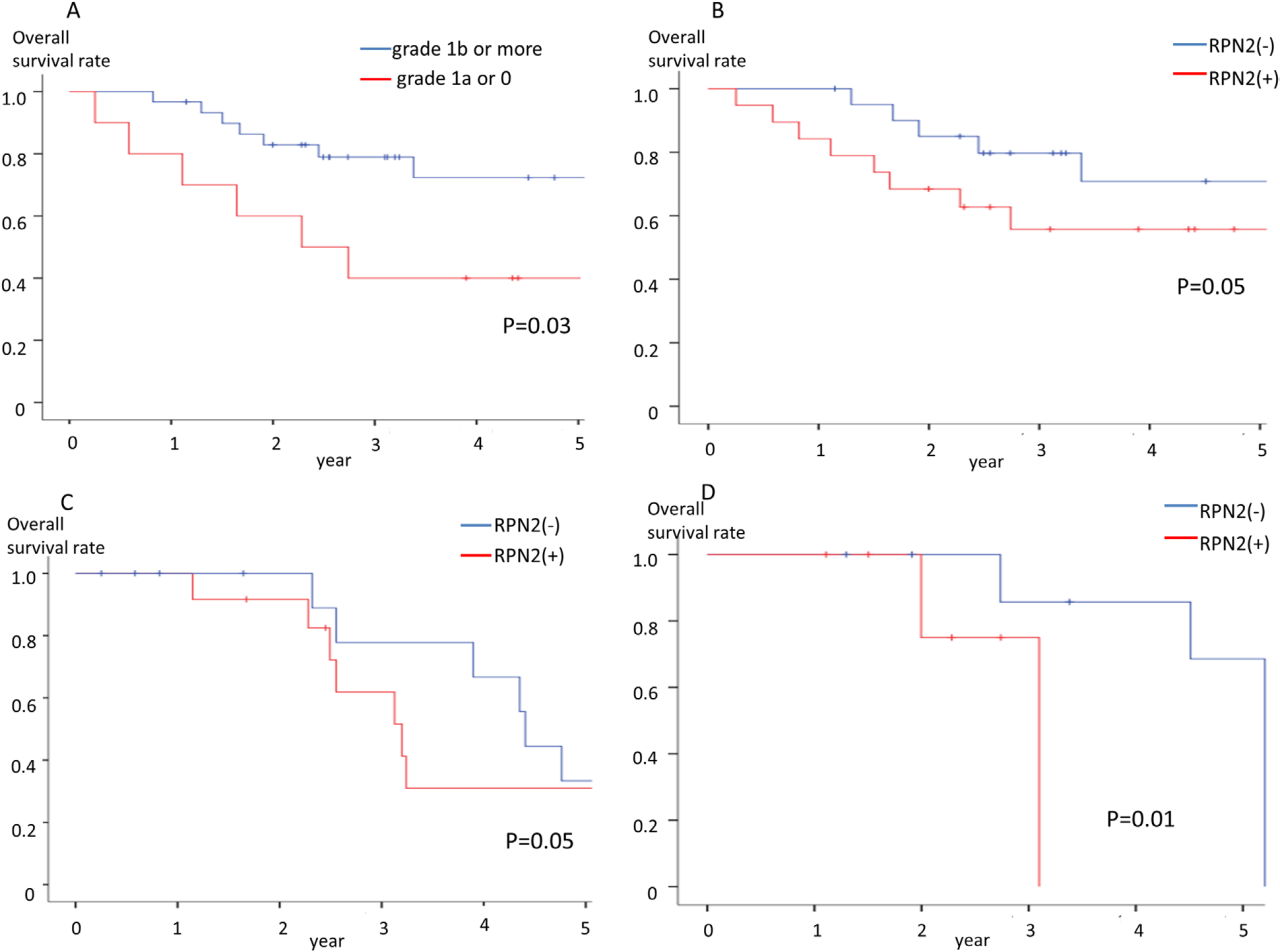
Overall survival rate **(A)** Relationship between Overall survival and histological response in advanced gastric cancer patients who were underwent docetaxel and cisplatin combination preoperative chemotherapy. **(B)** Relationship between Overall survival and RPN2 expression in advanced gastric cancer patients who were underwent docetaxel and cisplatin combination preoperative chemotherapy. **(C)** Relationship between Overall survival and RPN2 expression in clinical Stage II-III patients who were underwent docetaxel and cisplatin combination preoperative chemotherapy. **(D)** Relationship between Overall survival and RPN2 expression in clinical Stage IV patients who were underwent docetaxel and cisplatin combination preoperative chemotherapy.

**Figure 3:**
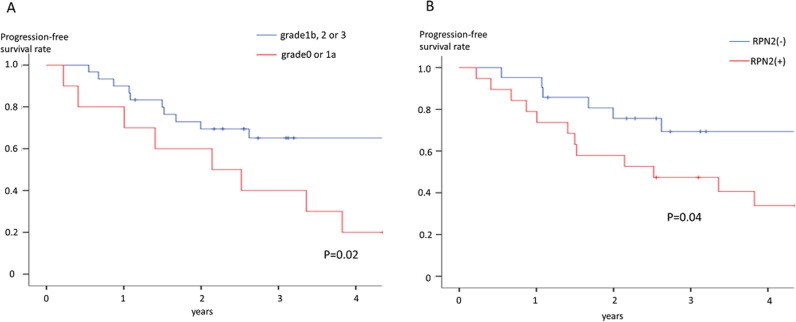
Progression-free survival rate **(A)** Relationship between Progression-free survival and histological response in advanced gastric cancer patients who were underwent docetaxel and cisplatin combination preoperative chemotherapy. **(B)** Relationship between Progression-free survival and RPN2 expression in advanced gastric cancer patients who were underwent docetaxel and cisplatin combination preoperative chemotherapy.

The advanced gastric cancer patients who were judged grade1b or more had significantly better overall survival and progression-free survival rate than the advanced gastric cancer patients who were judged grade1a or 0 by histopathological criteria (Figure 2A and 3A). Furthermore, the RPN2-negative group also had significantly better overall survival and progression-free survival rate than the RPN2-positive group (Figure 2B and 3B). As well as the above results, the RPN2-negative group had significantly better overall survival in clinical Stage II-III and Stage IV (Figure 2C and 2D).

### Knockout of RPN2 expression by genome editing increases sensitivity to docetaxel and cisplatin

**Figure 4:**
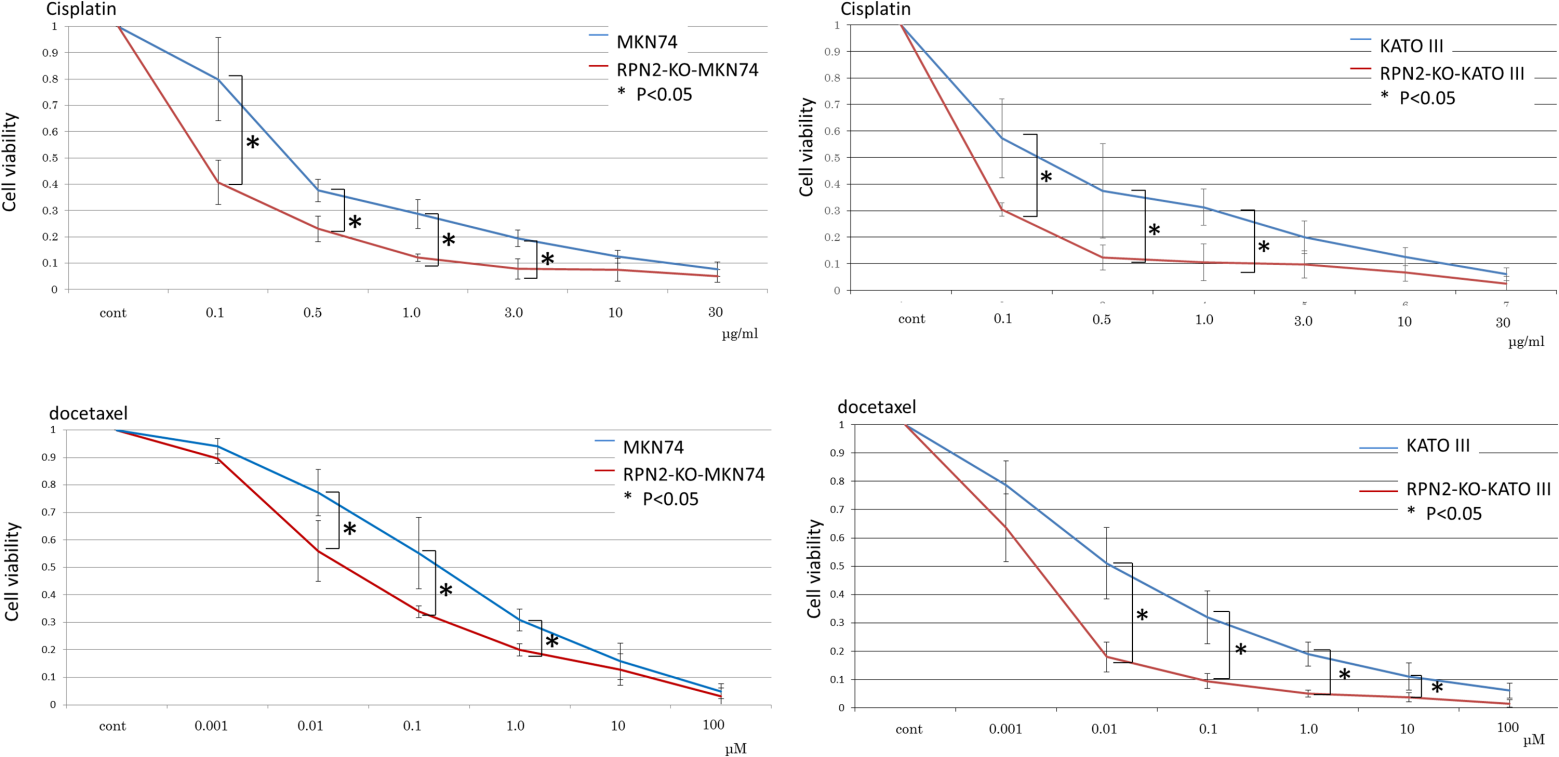
Knockout of RPN2 expression by genome editing enhances sensitivity to docetaxel and cisplatin Each control cells were transfected empty vector.

**Figure 5:**
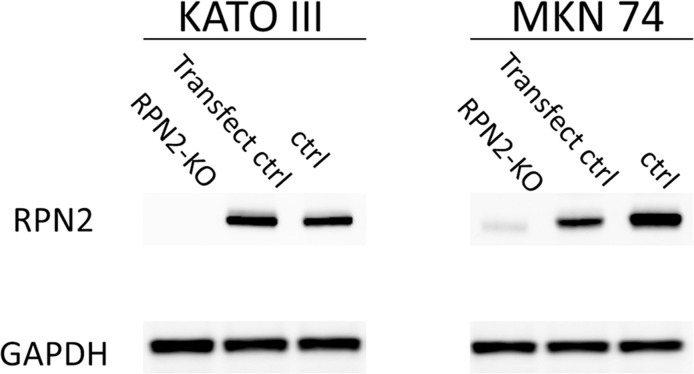
Western blot analysis of RPN2 expression in MKN74 and KATO III cells knocked out of RPN2 expression by genome editing The control and genome edited cells were collected and analyzed by western blotting with ant-RPN2 or anti-GAPDH antibody.

MKN74 and KATO III cells expressed RPN2 mRNA and protein [10]. We analyzed whether RPN2 knockout expression changed sensitivity to docetaxel and cisplatin. At 48 h after addition with docetaxel or cisplatin, there was significant cell death in knockout of RPN2 expression cell lines compared with control cell lines (Figure 4). And, we showed western blot analysis that MKN74 and KATO III cells expressed RPN2 and MKN74 and KATO III which were knocked out of RPN2 expression by genome editing did not express RPN2 (Figure 5). We found that expression of RPN2 had a significantly relationship with tolerance to docetaxel and cisplatin in gastric cancer cell lines.

### Effect of RPN2 to acquire the resistance of chemotherapy in gastric cancer

The main mechanism to confer drug resistance is the overexpression of adenosine triphosphate-binding cassette (ABC) transporters, which can raise efflux of anti-cancer drugs from cancer cells, accordingly reducing intracellular drug concentration. Three ABC transporters appear to account for most reported multi drug resistance in human: P-glycoprotein (P-gp), multidrug resistance-associated protein 1 (MRP1), and ATP-binding cassette sub-family G member 2 (ABCG2) [11]. We analyzed correlation between RPN2 and P-gp, ABCG2, or MRP1 expression in gastric cancer biopsy tissue in manner of immunohistochemical method. We confirmed a significantly correlation between RPN2 expression and P-gp expression in advanced gastric cancer biopsy tissue (Table 3). Subsequently, in advanced gastric cancer, we analyzed of factors associated with the effect of preoperative DCS chemotherapy. We found significantly correlation between Histological evaluation grade 1b over and sex, RPN2 expression or P-gp (Table 4). We were unable to find a significantly correlation between Histological evaluation grade 1b over and expression of ABCG2 or MRP1 (Table 4). In multivariate analysis, we confirmed that expression of RPN2 and P-gp were independent factors of prediction of preoperative chemotherapy effect in gastric cancer (Table 5).

**Table 3:** Correlation between RPN2 and P-gp, ABCG2, or MRP1 expression in gastric cancer

		RPN2 expression		P-Value
		Positive	Negative	
P-gp				
	Negative	1	20	< 0.05
	Positive	18	1	
ABCG2				
	Negative	13	14	= 0.906
	Positive	6	7	
MRP1				
	Negative	10	12	= 0.775
	Positive	9	9	

Abbreviations: RPN2 = ribophorin II; P-gp = P-glycoprotein; ABCG2 = ATP-binding cassette sub-family G member 2; MRP1 = Multidrug resistance-associated Protein 1.

Analyzed by Fisher's exact test.

**Table 4:** Univariate analysis of factors associated with the effect of preoperative chemotherapy for advanced gastric cancer

Factors		Histopathology		P-value
**grade 1b or over 29**	**grade 0 or 1a 11**	
Age	70≧	9	2	P = 0.416
	70<	20	9	
Sex	male	25	6	P = 0.032
	female	4	5	
cT	T3(SS)	6	1	P = 0.544
	T4a(SE)	22	10	
	T4b(SI)	1	0	
cN	N0	2	1	P = 0.573
	N1	7	5	
	N2	13	3	
	N3	7	2	
cM	present	17	9	P = 0.170
	absent	12	2	
cStage	IIB	1	2	P = 0.260
	IIIA	6	4	
	IIIB	6	3	
	IIIC	4	0	
	IV	12	2	
Macroscopic	Type 2	10	4	P = 0.417
type	Type 3	11	2	
	Type 4	8	5	
Histological	differentiated	9	2	P = 0.416
type	undifferentiated	20	9	
RPN2	Negative	20	1	P = 0.001
	Positive	9	10	
P-gp	Negative	19	9	P = 0.007
	Positive	10	2	
ABCG2	Negative	21	6	P = 0.281
	Positive	8	5	
MRP1	Negative	16	6	P = 0.972
	Positive	13	5	

Abbreviations: RPN2 = ribophorin II; P-gp = P-glycoprotein; ABCG2 = ATP-binding cassette sub-family G member 2; MRP1 = Multidrug resistance-associated Protein 1.

Analyzed by Fisher's exact test.

**Table 5:** Multivariate analysis of independent factors associated with the effect of preoperative chemotherapy for advanced gastric cancer

Factors	Odds Ratio (95% CI)	p value
Sex	1.302 (0.312–5.439)	P = 0.699
RPN2	3.750 (1.245–11.299)	P = 0.019
P-gp	0.825 (0.310–2.194)	P = 0.718

Abbreviations: RPN2 = ribophorin II; P-gp = P-glycoprotein.

## DISCUSSION

In this paper, we showed the clinical utility of RPN2 expression in endoscopic biopsy tissue for predicting tolerance to docetaxel and cisplatin combination chemotherapy. And, we also showed that RPN2 expression had a significantly relationship with tolerance to docetaxel and cisplatin in gastric cancer cell *in vitro*. We assessed effects of preoperative combination chemotherapy docetaxel and cisplatin using variety methods, including clinical imaging and pathological diagnosis.

Generically, cells have an important function as protecting the cell against noxious environment. The mechanisms as contributing to anticancer drug tolerance contain decreasing drug intake, increasing drug outflow, drug detoxification, initiation of anti-apoptotic factors, inhibition of pro-apoptotic factors, increasing DNA repair, and tolerance to DNA damage [12]. Of these, decreasing in the intracellular accumulation of hydrophobic chemotherapeutics due to members of the ABC transporter superfamily constitute a major mechanisms of drug resistance, and P-gp, that is one of the ABC transporter family, is an important molecule causing multidrug resistance in various cancer cells [13, 14] The silencing o RPN2 decreased the glycosylation and membrane localization of P-gp, and downregulation of RPN2 efficiently induced apoptosis in docetaxel-resistant human breast cancer cells in the presence of docetaxel [8]. Moreover, RPN2 silencing by shRNAs repressed lung tumor growth and sensitized the lung cancer cells to cisplatin treatment [9]. Other experiment showed that the expression of P-gp protein was decreased upon cisplatin treatment in RPN2-knockdown gastric cancer cell lines compared to control gastric cancer cell lines, and the viability of RPN2-knockdown gastric cancer cell lines were reduced relative to control gastric cancer cell lines in the presence of cisplatin [15]. We also showed correlation between RPN2 expression and P-gp expression in advanced gastric cancer tissue. Many advanced gastric cancer patients are underwent treatment of chemotherapy followed by gastrectomy, which has been accepted as a consensus therapy for advance gastric cancer in Japan. Fushida et al previously reported that the DCS regimen is a highly tolerable regimen as preoperative chemotherapy [5, 16–18]. Though significant improvement of prognosis has been made in this chemotherapy of advanced gastric cancer, but, survival rates were greatly decreased by the deficiency of response due to drug tolerance. So, we need to find new biomarkers that can be previously projected treatment effect in order to improve patient prognosis.

Gastric cancer patients undergo a routine examination of gastric mucosal biopsy under upper endoscopy. Immunohistochemical examination of biopsy tissues is a facility, safe and accessible method of judging biological features of neoplasm, so making individualized therapeutic plans possible. Inefficacious chemotherapy is not only unnecessary, but also baleful, so as side effect or losing chance of curative resection, in the preoperative chemotherapy. Thus, the predictive factors for chemotherapy response are immediately needed. There are some reports described predictive factors for therapeutic responses to docetaxel or cisplatin. Increased expression of Excision repair cross-complementation group 1 (ERCC1) correlated with a better prognosis of cervical cancer and ERCC1 expression was a useful predictive marker of locally advanced squamous cell carcinoma of the head and neck in patients treated with cisplatin [19, 20]. Hwang et al, reported that the median progression-free survival of the high level Class III β-tubulin (TUBB3) expression patients, who were with advanced gastric cancer underwent first line chemotherapy docetaxel and cisplatin or paclitaxel and cisplatin, were significantly shorter than the low-level TUBB3 expression patients, but overall survival was not associated with TUBB3 expression [21]. Moreover, ERCC1 had no clinical effect on progression-free survival or overall survival in gastric cancer patients [21]. In advanced gastric cancer, there is not yet a utility predictive biomarker, which is associated with progression-free survival and overall survival, for sensitivity to docetaxel and cisplatin combination chemotherapy.

Our study showed that there was a significant difference in overall survival and progression-free survival not only between RPN2 positive and RPN2 negative groups, but also between judged as grade1b or more and judged grade as 0 or 1a by histopathological criteria. And, we showed that significant association between RPN2 expression and effects to chemotherapy were found. Also *in vitro*, we indicated that RPN2 expression reflected docetaxel and cisplatin sensitivity in gastric cancer cell lines. This report shows the possible use of RPN2 expression as a predictive biomarker for docetaxel and cisplatin combination preoperative chemotherapy in advanced gastric cancer for the first time. We believe that the following strategies will be established. If RPN2 expression cannot be observed in advanced gastric cancer biopsy tissue, DCS regimen will be performed with preoperative chemotherapy. And, if RPN2 expression can be detected, other regimen without docetaxel and cisplatin will be performed, for example S-1 and oxaliplatin.

## MATERIALS AND METHODS

### Patients and samples

We retrospectively used 40 paraffin-embedded tumor samples endoscopically biopsied from advanced gastric cancer patients, who were Eastern Cooperative Oncology Group performance status 0–1, received no prior chemotherapy, radiotherapy, or major surgical procedure and provision of signed written informed consent, before treatment with the DCS therapy, consisted of docetaxel 35 mg/m^2^ and cisplatin 35 mg/m^2^ as an intravenous infusion on Days 1 and 15, and S-1 administered at a dose of 80 mg/m^2^/day divided into two split daily doses for 14 days, followed by 14 days of rest, at University of Fukui Hospital from 2008 to 2014 [5]. Before treatment, all patients underwent upper endoscopy, gastrography, and enhanced CT imaging from abdomen to pelvic for staging according to the Japanese classification of gastric carcinoma: 3^rd^ edition [22]. After being diagnosed with advanced gastric cancer, all patients received two cycles of induction DCS chemotherapy. Imaging by upper endoscopy, CT, and gastrography was conducted in all patients post chemotherapy. After 2 cycles of chemotherapy, 31 patients underwent gastrectomy with D2 resection, 9 patients continued DCS chemotherapy and subsequent gastrectomy with D2 resection. Clinical data are summarized in Table 1. Thirty six patients underwent curative gastrectomy (R0). And other 4 patients underwent non curative gastrectomy (R1), because they were diagnosed peritoneal cytology positive for carcinoma cells. All patients were given post-operative chemotherapy of S-1 for a year.

This study was confirmed by the institutional review board of University of Fukui, Faculty of Medical Sciences. Written informed consent was got from all patients.

### Assessment of clinical response to preoperative chemotherapy

We judged clinical effect to DCS chemotherapy by the Response Evaluation Criteria in Solid Tumors (RECIST) v1.1 and histopathological criteria. All patients underwent gastrectomy with D2 resection and histopathological tumor regression from preoperative chemotherapy was measured by judging the resected tumors pursant to a three-grade score established by the Japanese Guidelines for the Clinical and Pathological Studies on gastric cancer [22], with histopathological effects classified into four categories, from 0 to 3 (Supplementary Table 1)

### Immunohistochemical staining

Paraffin sections, 4μm thick, were de-paraffinized with xylene and dehydrate through a grade ethanol series. Endogenous activity was blocked by incubation for 30 min with 1% hydrogen peroxidase methanol. These hydrate sections were incubated in dilution of skim milk powder for 30 min to reduce non-specific staining, and incubated overnight with anti-RPN2 Ab (Aviva systems Biology, CA), ant-P-gp Ab (Abcam, CA), anti-ABCG2 Ab (Santa Cruz Biotechnology, TX), or anti-MRP1 Ab (Abcam) at 4°C in humidified chamber. After washing with Tris-buffered saline (TBS) buffer, and analyzed for the expression of RPN2 protein by the ChemMate method (Dako, Denmark). The sections were developed with activated 3′-diaminobenzidinetetrahydrochloride for 5 min and the reaction was stopped in TBS. Finally, the slides were lightly counterstained with hematoxylin. The expression was interpreted as positive when the protein was expressed in >20% of cancer cells using ImageJ software (http://rsb.info.nih.gov/ij/).

### Cell culture

Human gastric cancer cell lines MKN74 and KATO III were obtained from JCRB Cell Bank. We had established RPN2 knockout MKN74 (RPN2-KO-MKN74) and RPN2 knockout KATO III (RPN2-KO-KATO III) cell lines by genome editing [10]. All cells were cultured in RPMI 1640 (Sigma-Aldrich, MO) supplemented with 10% foetal bovine serum (Gibco, CA), 100 U/ml penicillin, and 100 μg/ml streptomycin at 37°C with 5% CO_2_ incubation.

### Western blot

Total cell protein was extracted using RIPA buffer. Proteins in the lysate were resolved by SDS-PAGE using a 5-20% SuperSep gel (Wako, Japan). The resolved proteins were transferred to nitrocellulose membrane. Protein bands were incubated with primary antibody overnight at 4°C. Signals were visualized by enhanced chemiluminescence according to the manufacturer's instructions (GE Healthcare). Anti-RPN2 Ab was from AVIVA SYSTEMS and anti-GAPDH Ab was from Abcam.

### Chemotherapy sensitivity assay

To judge the force of RPN2 on docetaxel and cisplatin (Wako) sensitivity in gastric cancer cell, 1 × 10^5^ cells were seeded into 12-well plates (Corning, NY). Cells were treated with docetaxel at increasing concentrations (0.001, 0.01, 0.1, 1.0, 10 and 100 μM) for 48 h, and cisplatin at increasing concentrations (0.1, 0.5, 1.0, 3.0, 10 and 30 μg) for 48 h. The cell survival rate was determined using the cell counting kit CCK-8 (Dojin Laboratories, Japan). The color intensity was determined at 450 nm.

### Statistical analysis

Statistically significant differences in clinical, pathological immunohistochemical expression findings were assessed by cross-tabulation, and statistical evaluations were determined by the χ^2^ test using SPSS software (IBM Corp., IL). And, Multivariate analysis was performed using the logistic regression model. Logistics regression was used to calculate Odds Ratios and 95% confidence intervals (95% CIs) after controlling simultaneously for potential confounders.

Overall survival was measured from the data of stating initial treatment to the date of death, or last follow-up. Progression-free survival was measured from the date of starting initial treatment to the date of first evidence of relapse or death due to any cause. For patients who had not relapsed or died, progression-free survival was censored at the last date at which the absence of relapse was confirmed. Overall and progression-free survival curves were calculated by the Kaplan-Meier method. The outcomes from different groups of patients were compared by log-rank test using SPSS software. P-values <0.05 were considered statistically significant.

## CONCLUSION

Finally, RPN2 expression of upper endoscopic biopsy tissues can be predictive biomarker of sensitivity to docetaxel and cisplatin combination preoperative chemotherapy in advanced gastric cancer patients. Although a large-scale clinical trial is necessary to make a final conclusion, the new knowledge in this study is important clinical deduction for advanced gastric cancer patients planning to undergo preoperative chemotherapy.

## SUPPLEMENTARY MATERIALS TABLE



## Abbreviations

ABC: adenosine triphosphate-binding cassette
ABCG2: ATP-binding cassette sub-family G member 2
CR: complete response
CT: computed tomography
DCS: docetaxel cisplatin and S-1
ERCC1: Excision repair cross-complementation group 1
MRP1: multidrug resistance protein 1
PD: Progressive Disease
P-gp: P-glycoprotein
RECIST: Response Evaluation Criteria in Solid Tumors
PR: Partial Response
RPN2: Ribophorin II
SD: Stale Disease
sh: small hairpin
TBS: Tris-buffered saline
TUBB3: Class III β-tubulin
