# Supplementary material for: RPN2 is effective biomarker to predict the outcome of combined chemotherapy docetaxel and cisplatin for advanced gastric cancer
Source: Oncotarget. 2018 Mar 8;9(20):15208–18. doi: 10.18632/oncotarget.24622 (PMC5880597; doi:10.18632/oncotarget.24622)
Supplement: Supplementary file 1 [file oncotarget-09-15208-s001.pdf]

RPN2 is effective biomarker to predict the outcome of combined chemotherapy docetaxel and cisplatin for advanced gastric cancer

SUPPLEMENTARY MATERIALS

Supplementary Table 1: Histological evaluation criteria of tumor response after preoperative therapy

Grade0	(no effect)	No evidence of effect.
Grade1	(slight effect)	
	Grade1a (very slight effect)	Viable tumor cells occupy more than 2/3 of the tumorous area.
	Grade1b (slight effect)	Viable tumor cells remain in more than 1/3 but less than 2/3 of the tumorous area.
Grade2 (considerable effect)		Viable tumor cells remain in less than 1/3 of the tumorous area.
Grade3 (complete response)		No viable tumor cells remain. It is recommended that the finding is confirmed on additional sectioning.
